# Editorial: Genome editing technology in polyploid crops

**DOI:** 10.3389/fpls.2023.1340455

**Published:** 2023-12-11

**Authors:** Chao Li, Yu Shu, Qiong Hu

**Affiliations:** Oil Crops Research Institute, Chinese Academy of Agricultural Sciences, Key Laboratory for Biology and Genetic Improvement of Oil Crops, Ministry of Agriculture and Rural Affairs, Wuhan, China

**Keywords:** polyploid crops, genome editing tools, crop genetic improvement, genetic transformation, tissue culture free

Polyploid crops are of great importance, as they make up a substantial proportion of the world’s primary food and cash crops. Polyploidy can be classified into two types: autopolyploidy, which involves the duplication of a single genome, and allopolyploidy, which involves the combination of two or more different genomes to give rise to a new species. The increased genetic material in polyploid crops can lead to enhanced agronomic traits and improved productivity, contributing to food security and crop resilience. Advancements in Next-Generation Sequencing (NGS) technology and bioinformatics tools have significantly expanded the availability of genetic information for functional studies of polyploid genomes. Effective use of genomic information to create genetic variation and novel germplasm resources for crop genetic improvement is a major challenge in the post-genomic era.

Following the initial demonstrations of the efficacy of genome modification in mammalian cells using the Cas9 nuclease (Cong et al., 2013), there has been significant growth in the development and application of CRISPR–Cas genome editing tools specifically tailored for crop plant genome editing. The CRISPR-based genome editing system possesses a distinct advantage in the assembly of multiplexed gRNA cassettes, making it particularly suitable for the simultaneous gene modification of multiple gene copies in the genomes of polyploid crops. This Research Topic aims to investigate the potential applications of CRISPR–Cas technology in genome editing of polyploid crops and showcases several original research papers that explore different aspects of this field.

The establishment of a highly efficient genome editing platform greatly depends on effective CRISPR/Cas delivery methods. The use of agrobacterium-mediated T-DNA transformation remains the most commonly employed approach to generating transgenic plants. One article featured in this Research Topic presents an improved CRISPR/Cas9-based transformation protocol in highbush blueberry, a polyploid woody species, through the utilization of *de novo* shoot organogenesis (Vaia et al.). Similarly, to achieve genotype-independent transformation, a comparable strategy has been implemented in *Brassica napus*, utilizing epicotyl and higher stem (internodal) segments as recipient material (Chu et al., 2020). Furthermore, the utilization of regeneration-related genes, such as *WUSCHEL* (*WUS2*), *BABY BOOM* (*BBM*), and *SHOOT MERISTEMLESS* (*STM*), holds great promise for substantially improving the efficiency of plant transformation (Lowe et al., 2016). A CRISPR-Combo platform has been developed to accelerate plant regeneration and generate genome-edited plants by combining Cas nucleases for genome modification with gene activation systems to activate morphogenic genes, such as *WUS* and *WOX11* (Pan et al., 2022).

The use of engineered virus vectors to deliver genome-editing reagents has been regarded as a viable method to circumvent tissue culture-based transformation methods. However, the limited packaging capacity of the majority of plant virus vectors, typically in the range of 1-2 kb, hinders the efficient delivery of larger CRISPR-Cas9 cassettes (>4.5 kb). The effectiveness of Sonchus yellow net rhabdovirus (SYNV), a plant negative-strand RNA virus, has been validated for the delivery of the complete CRISPR-Cas9 cassette (Ma et al., 2020). High-frequency genome mutagenesis has been demonstrated in infected somatic cells of allotetraploid tobacco. However, the fact that plant viruses are unable to infect meristematic or germline cells has posed a limitation on the transmission of these mutations to the next generation. It has been demonstrated that the fusion of gRNA with the mobile *Flowering Locus T* (*FT*) RNA sequence enhances the efficiency of mutation induction in meristematic cells, resulting in the generation of heritable mutations (Ellison et al., 2020). The article included in this Research Topic describes the utilization of Cotton Leaf Crumple Virus (CLCrV) as a vehicle to deliver gRNA and *FT* RNA-fused gRNA in allotetraploid cotton (Lei et al.). Data from the study indicated that the use of CLCrV was effective in delivering gRNA and FT-gRNA into plant cells, leading to mutagenesis in Cas9-overexpressed cotton. However, despite the successful delivery, the study found it difficult to detect heritable mutant progeny in cotton plants infected with the FT-gRNA virus vector (Lei et al.). Thus, although FT has been utilized as a mobile sequence to guide the migration of gRNA to meristematic cells, further optimization may still be required in certain polyploid crop species.

Due to the strong capability of CRISPR/Cas tools to induce precise genome modifications, they have been widely employed in various scenarios for the genetic improvement of polyploidy crops. While many studies tend to focus on generating single or multiple mutations within the coding region of genes, leading to noticeable phenotypic variation, it should be noted that numerous important agronomic traits are controlled by quantitative trait loci (QTLs), such as yield components, growth stage duration, and quality traits. Hence, it is crucial to consider the impact of QTLs in comprehensive trait improvement strategies. Genome editing of cis-regulatory elements has proven to be an effective approach to regenerating domestication QTLs. By precisely modifying these regulatory regions, it becomes possible to assess the impact of continuous cis-regulatory variation on quantitative traits (Rodríguez-Leal et al., 2017). In this Research Topic, one article presents evidence for the successful use of CRISPR/Cas9 to modify the promoter sequence of the *VERNALIZATION 1* gene (*VRN-1*), a key regulator of floral initiation, in plants of the “Chinese Spring” variety. This targeted modification resulted in the generation of new VRN-A1 variants. Minor changes in the promoter sequence did not impact heading time, but an 8 bp deletion within the −125 to −117 bp region of the promoter shortened head emergence time by 2-3 days (Miroshnichenko et al.). In addition, genome editing of upstream open reading frames (uORFs) has been used to precisely manipulate gene translation and create a wide range of variation in crop plants (Xing et al., 2020).

In conclusion, the advancement of genome editing technologies in polyploid plants has efficiently facilitated the creation and utilization of variation, thereby opening up new possibilities to revolutionize agriculture through next-generation breeding strategies. In recent years, genome editing techniques have gained widespread adoption in many major polyploid plants. However, the complexity of polyploid plant genomes necessitates further refinement and development of the genome editing toolbox. For instance, the editing efficiency of adenine base editors (ABEs) and primer editors still requires optimization in most dicotyledonous polyploid crops. Additionally, agrobacterium-mediated T-DNA transformation is the predominant method used to deliver genome editing reagents to these crops. However, the recalcitrant nature of many elite commercial varieties presents challenges to achieving successful genetic transformation and genome editing. Establishing tissue-culture-free and haploid-inducer-mediated genome editing is an effective strategy to overcome the limitations faced by polyploid crops.

## Author contributions

CL: Writing – original draft, Writing – review & editing. YS: Writing – review & editing. QH: Writing – review & editing.
